# New Books

**Published:** 2004-11

**Algorithms in Bioinformatics**

Inge Jonassen, Junhyong Kim, eds.

New York:Springer-Verlag, 2004. 476 pp.

ISBN: 3-540-23018-1, $84

**Antisense Therapeutics, 2nd ed.**

M. Ian Phillips

Totowa, NJ:Humana Press, 2004. 344 pp.

ISBN: 1-58829-205-3, $125

**Assembling the Tree of Life**

Joel Cracraft, Michael J. Donoghue

New York:Oxford University Press, 2004. 592 pp.

ISBN: 0-19-517234-5, $59.95

**Bioinformatics: A Practical Guide to the Analysis of Genes and Proteins, 3rd ed.**

Andreas D. Baxevanis, B.F. Francis Ouellette, Mark Boguski, Gerard Bouffard, Stephen Bryant, Barbara Butler, Sam Cartinhour, Colombe Chappey, Mark Hershkovitz, Christopher Hogue, Jonathan Kans, David Landsman, Detlef Leipe, James Ostell, Greg Schuler

Hoboken, NJ:John Wiley & Sons, 2004. 512 pp.

ISBN: 0-471-47878-4, $79.95

**Cancer Gene Therapy**

David T. Curiel, Joanne T. Douglas

Totowa, NJ:Humana Press, 2004. 504 pp.

ISBN: 1-58829-213-4, $165

**Epigenetics Protocols**

Trygve O. Tollefsbol

Totowa, NJ:Humana Press, 2004. 320 pp.

ISBN: 1-58829-336-x, $99.50

**Gene Genealogies, Variation and Evolution: A Primer in Coalescent Theory**

Jotun Hein, Mikkel Schierup, Carsten Wiuf

New York:Oxford University Press, 2004. 350 pp.

ISBN: 0-19-852995-3, $124.50

**Genome Transcriptome and Proteome Analysis**

Alain Bernot

Hoboken, NJ:John Wiley & Sons, 2004. 240 pp.

ISBN: 0-470-84954-1, $115

**Guide to Mutation Detection**

Graham R. Taylor, Ian N. Day

Hoboken, NJ:John Wiley & Sons, 2004. 352 pp.

ISBN: 0-471-23444-3, $89.95

**Mobile Genetic Elements: Protocols and Genomic Applications**

Wolfgang J. Miller, Pierre Capy

Totowa, NJ:Humana Press, 2004. 304 pp.

ISBN: 1-58829-007-7, $89.50

**Modular Protein Domains**

Giovanni Cesareni, Marlo Gimona, Martus Sudol, Michael Yaffe, eds.

Totowa, NJ:Humana Press, 2004. 524 pp.

ISBN: 3-527-30813-X, $185

**Oligonucleotide Synthesis**

Piet Herdewijn

Totowa, NJ:Humana Press, 2004. 456 pp.

ISBN: 1-58829-233-9, $125

**Protein Synthesis and Ribosome Structure: Translating the Genome**

Knud H. Nierhaus, Daniel N. Wilson

Hoboken, NJ:John Wiley & Sons, 2004. 592 pp.

ISBN: 3-527-30638-2, $180

**Statistical Methods in Molecular Evolution**

Rasmus Nielsen, ed.

New York:Springer-Verlag, 2004. 520 pp.

ISBN: 0-387-22333-9, $89.95

**Stroke Genomics: Methods and Reviews**

Simon J. Read, David Virley

Totowa, NJ:Humana Press, 2004. 352 pp.

ISBN: 1-58829-333-5, $125

**Thompson & Thompson Genetics in Medicine, Revised**

Robert Nussbaum, Roderick McInnes, Huntington Willard

New York:Springer-Verlag, 2004. 540 pp.

ISBN: 0-7216-0244-4, $49.95

**The Proteus Effect: Stem Cells and Their Promise**

Ann B. Parson

Washington, DC:National Academies Press, 2004. 256 pp.

ISBN: 0-309-08988-3, $24.95

**Transcription Factors**

Manfred Gossen, Jörg Kaufmann, Steven J. Triezenberg, eds.

New York:Springer-Verlag, 2004. 581 pp.

ISBN: 3-540-21095-4, $399

**Understanding DNA: The Molecule and How it Works**

Chris Calladine, Horace Drew, Ben Luisi, Andrew Travers

New York:Springer-Verlag, 2004. 352 pp.

ISBN: 0-12-155089-3, $45

**Welcome to the Genome: A User’s Guide to the Genetic Past, Present, and Future**

Rob DeSalle

Hoboken, NJ:John Wiley & Sons, 2004. 240 pp.

ISBN: 0-471-45331-5, $29.95

